# 

**Published:** 1919-06

**Authors:** 


### Old Jokes That We Have Met.

Counting The Cost:—Patience—Have you been vaccinated lately?

Patience—Yes.

"Does it cost much,"

"Does it? Why, say, Fred couldn't hug me for over three weeks?"

---

"An' phwat did yez say was the doctor's name?" he asked the nurse.

"Dr. Kilpatrick."

"Thot settles ut." The patient twisted uneasily. "He won't operate on me."

"But he's very skillful," argued the nurse.

"That's as may be," firmly met her, "but me name happens to be Patrick."—Ex.

---

Feminine Diplomacy—Young Physician—But isn't seven dollars a week rather high for a room like this?

Landlady—Oh, no; not for a doctor.

Young Physician—And why not for a doctor, pray?

Landlady—Well, you see, this is a very unhealthy house, and there is never a week passes but a dozen or more of my roomers are ill.

---

Young Doctor—That patient has St. Vitus' dance, hasn't he?

Old Doctor—No. He has "the buzz-buggy twitch," caused by dodging autos.

---

Scrappers, But.—Colonel (of a very gallant Canadian regiment)—"Now, boys, here's the English general coming to inspect you. Keep steady, no spitting, and, for Heaven's sake, don't call me Alf."

---

Too Much.—Judge. Now, sir, tell us about your martial relations—were they pleasant?

Bilback. Pleasant enough, your honor. But they wanted to troops in France, the Lancet says: "Only one sign has been live on me all the time.

